# Junction investigation of graphene/silicon Schottky diodes

**DOI:** 10.1186/1556-276X-7-302

**Published:** 2012-06-11

**Authors:** Muatez Mohammed, Zhongrui Li, Jingbiao Cui, Tar-pin Chen

**Affiliations:** 1 Department of Physics and Astronomy, University of Arkansas at Little Rock, Little Rock, AR, 870-71656, USA

**Keywords:** Graphene, heterojunction, Schottky diode

## Abstract

Here we present a facile technique for the large-scale production of few-layer graphene flakes. The as-sonicated, supernatant, and sediment of the graphene product were respectively sprayed onto different types of silicon wafers. It was found that all devices exhibited current rectification properties, and the supernatant graphene devices have the best performance. Schottky junctions formed between graphene flakes and silicon n-type substrates exhibit good photovoltaic conversion efficiency while graphene/p-Si devices have poor light harvesting capability.

## Background

Since the first demonstration of graphene nanosheets in 2004, it has been considered as a chemically stable and mechanically strong new material [1]. Tremendous work has been devoted to this material because it has exhibited outstanding properties particularly in the optoelectronic field [2]. The two dimensional honeycomb lattice of the graphene leads to a hybridization of sp^2^ which, in turn, leads to extraordinary electrical properties with ultrahigh carrier mobility (approximately 100,000 cm^2^/Vs) [3]. A monolayer of graphene has a thickness of 0.34 nm and absorbs 2.3 % of white light [4]; even graphene layers of 1,000 nm thick still have a transparency of approximately 70 % [5], which makes it possible to use graphene as transparent electrodes [6]. Graphene has the advantages over carbon nanotubes of being naturally compatible with thin film processing, enabling large device areas and hence, high operating powers. Also, graphene is more readily scalable and has a lower contact resistance. The combination of these enticing electrical and optical properties of the graphene motivated the researchers to experience it in the field of optoelectronic devices [7].

Previous use of graphene in organic solar cell applications was mainly confined as flexible transparent electrodes to replace transparent indium tin oxide or fluorine-doped tin oxide for collecting charge carriers [8]. Recently, graphene-on-silicon configurations were made into solar cells by using membrane transfer technique [9]. However, the fabrication process based on membrane transfer is expensive and difficult to scale up. The previous studies have explored electron transport in graphene; the Schottky barriers between graphene and silicon have not been studied thoroughly. The Schottky barriers have been observed at bulk, highly ordered pyrolytic graphite/silicon interfaces [10], but no photocurrents could be measured; a comparison of n- and p-type substrates was not given in this prior work. Moreover, the local effect of light absorption on the *J-V* characteristics of graphene/silicon interfaces has not been studied.

In this work we present a facile technique for the large-scale production of few-layer graphene flakes. Next, as-sonicated (So), supernatant (Su) and sediment (Se) of the graphene product were respectively sprayed onto n-/p-type silicon wafers. The current rectification properties of the formed Schottky junctions between different graphene sources and silicon substrates were compared.

## Methods

The graphene flakes were synthesized through chemical vapor deposition of acetylene on a MgO-supported Fe-Co bimetallic catalyst (Fe-Co/MgO with a stoichiometric composition of 2.5:2.5:95 wt%) [11]. The catalyst was prepared by using the impregnation technique. Initially, the weighted amounts of Fe(NO_3_)_3_·9H_2_O and Co(NO_3_)_2_·6H_2_O were dissolved in ethanol under agitation. Subsequently, the MgO powder with a surface area of 130 m^2^/g was mixed the solution and followed by drying at 60°C overnight. The catalyst was obtained by calcinating the resulting mixture in air at 500°C for 2 h. Graphene sheets can grow on the catalyst system from pyrolysis of acetylene at 1,000°C with the argon flow as carrier gas. The mixture product can be collected after 30 min of reaction and cooled under argon flow for about 10 min. Impurities like catalystsupport MgO and Fe-Co metal particles can be removed by washing the mixture product with hydrochloric acid under sonication. The purified graphene sheets can be obtained after filtration and washing.

**Figure 1:**
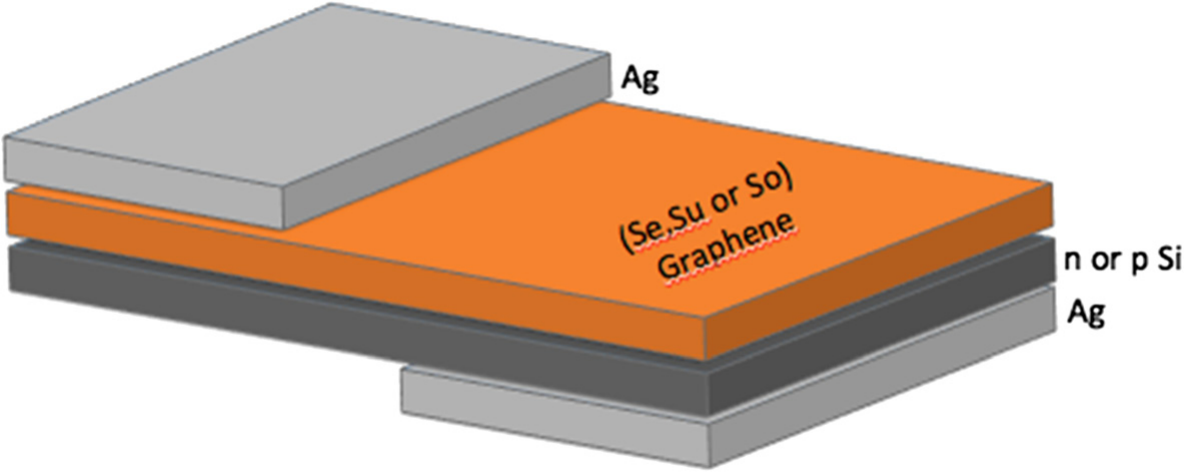
**Schematic diagram of a graphene-on-silicon device.** Characterization techniques.

Purified graphene flakes were first dispersed in pure *N*-methyl-2-pyrrolidone (NMP, 0.1 mg/mL) under sonication(So, G-NMP). The G-NMP solution can be directly utilized to make G/n-Si devices. Part of the G-NMP solution was centrifuged; the supernatant (Su) and the sediment (Se) were taken out respectively and used for the fabrication of G/n-Si devices. The three different solutions were sprayed respectively on n-type silicon wafer (resistivity, 0.295 Ohm·cm and mobility, 1,026 cm^2^/Vs), p-type silicon substrate (resistivity, 21 Ohm·cm and mobility, 1,502 cm^2^/Vs) and glass substrates (for reference) by airbrushing. A silicon wafer with a window of pre-deposited insulating layer and a glass substrate were placed on a heating platform side by side (so that the resulting graphene flake films on Si and glass substrates should have the same thickness) and heated up to 150°C in order to evaporate the NMP solvent from the coatings. The schematic diagram of graphene-on-silicon Schottky device was displayed in Figure 1.

To characterize the morphological properties of graphene nanosheets, several techniques such as microscopy and X-ray diffraction (XRD) were utilized. Atomic force microscopy (AFM) images were obtained on Veeco Dimension 3100 AFM system (Veeco Instruments, Inc., NY, USA). Scanning electron microscopy (SEM) images were obtained using a JEOL 7000 F high-resolution scanning electron microscope (JEOL Ltd., Tokyo, Japan). This microscope has a resolution of 1.2 nm at an accelerating voltage of 15 kV and a working distance of 10 mm. The final products were mounted on aluminum pins with double-sided carbon tape and their corresponding SEM images were obtained. Elemental analysis was performed with Genesis energy dispersive spectrometer system (EDAX Inc., Mahwah, NJ, USA). The X-ray powder diffraction profiles of graphene sheets were recorded in θ-2θ mode on Bruker D8 Discovery diffraction system (Bruker AXS Corporation, Madison, WI, USA). The monochromatic Cu *Kα* radiation line and general area detector diffraction system were used as an excitation source and detector, respectively. The experiments were carried out in Bragg-Brentano geometry.

To understand how a different graphene source affect the G/n-Si Schottky junction properties, current density-voltage (*J-V*) characteristics were investigated in the dark and under illumination using a solar simulator at air mass coefficient 1.5 (approximately 100 mW/cm^2^) inside a glove box in a nitrogen environment. The illumination was on the graphene flake side. The devices were irradiated in an area of 1 × 1 cm^2^ and data were recorded using a Keithley 2400 source meter (Keithley Instruments Inc., Cleveland, OH, USA).

## Results and discussions

**Figure 2:**
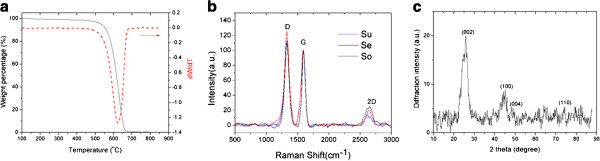
**TGA result, Raman spectra, and XRD pattern of the graphene flakes.** The TGA result (**a**), Raman spectra (**b**) and XRD pattern (**c**) of the graphene flakes obtained from the supernatant, sonication, and sediment.

As shown Figure 2a, the thermal gravimetric analysis (TGA) results show the purity of graphene flake as-purified sample is better than 99 wt%. The combustion temperature is around 620°C. Raman spectroscopy is a nondestructive optical technique, has been successfully used to characterize graphene and other carbon-based materials [12,13]. Furthermore, it was shown that the Raman spectrum of graphene provides useful information about its crystallinity and the number of layers present within the sample [4]. The typical Raman spectra (Figure 2b) of the few-layer graphene has three main peaks which are commonly referred to as the *D* band (approximately 1,350 cm^−1^), *G* band (approximately 1,580 cm^−1^) and the 2*D* band (approximately 2,650 cm^−1^). The *D* band arises due to the breathing modes of sp^2^ atoms in rings and its intensity is usually associated with defects in the carbon-based material [14], in the case of graphene, a substantial contribution which typically comes from the edge effects [15]. The *G* band is often referred to the E_2g_ mode at the Brillouin zone center due to the bond stretching of sp^2^ atoms in both ring and chains [16]. The 2*D* band is the second order mode and its shape as well as its position is used to identify a single layer from bilayer and few (less than five) layer graphene [4]. Figure 2b shows the Raman spectrum of our graphene flakes measured at 633 nm excitation.

The crystallinity and number of layers in the graphene nanosheets can be analyzed by XRD technique. The XRD profile of the graphene sheets grown by radio frequency catalytic chemical vapor deposition method was shown in Figure 2c. The typical features for graphite at 25.3° (002) and 49.1° (004) are identified in this graphene XRD pattern [17]. The 44.7° (100) and 74.7° (110) diffraction peaks originate from the two-dimensional in-plane symmetry along the graphene sheets [18]. The layer-to-layer distance (*d*-spacing) between two subsequent graphene sheets can be calculated from the (002) diffraction peak position [17]. The width of the diffraction peak can be used to evaluate the crystallite size by using the Scherrer equation (thickness = 0.9 λ/(*B* cos θ), where λ is the x-ray wavelength, *B* is the full width at half maximum of the diffraction peak, and θ is the Bragg angle) [19]. Based on the values of the *d*-spacing and the size of crystallite, the graphene sheets in this work were estimated to have in average of about four layers.

**Figure 3:**
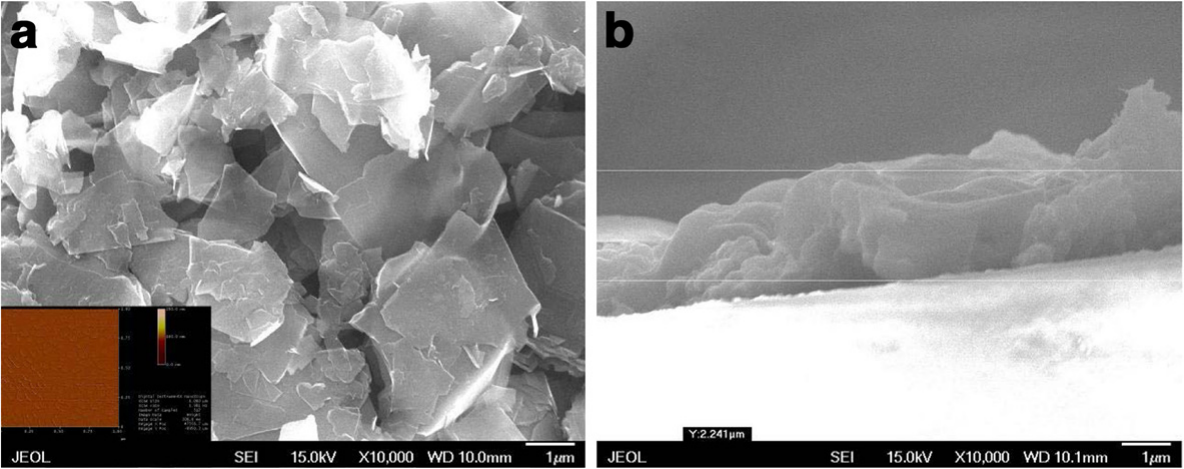
**SEM and AFM images of typical graphene-on-silicon devices.** SEM top view (**a**) and cross section view (**b**) images of the typical graphene-on-silicon devices. The inset in (a) displays the AFM image of the graphene flakes obtained from the supernatant.

The top view morphologies of a typical G/Si device made of supernatant were displayed in SEM and AFM images (Figure 3). Both SEM and AFM characterization on the graphene coating reveals that graphene flakes are overlapped and interconnected, which ensures a conducting pathway even if there are cracks formed in one of the layers.

**Figure 4:**
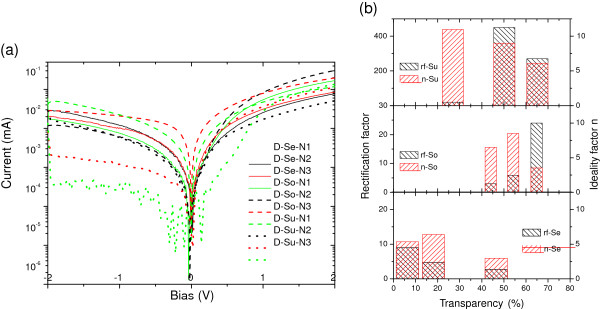
***J-V*characteristic curves of G/n-Si devices, rectification and ideality factors.** (**a**) The *J-V* characteristic curves of G/n-Si devices were collected in dark. (**b**) The rectification factors (left y-axis) and the ideality factors (right y-axis) of G/n-Si devices with different graphene source and different thickness.

**Figure 5:**
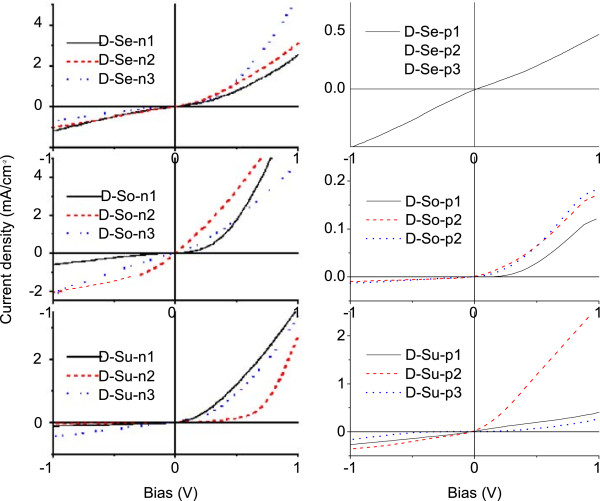
***J-V*characteristic curves of G/n-Si and G/p-Si devices with different graphene sources in the dark.**

The typical dark current–voltage characteristic of G/n-Si heterojunction device is displayed in the top inset of Figure 4. The G/n-Si heterojunctions are highly rectifying with an ‘on/off’ current ratio of 10 to approximately 10^3^ at ±1 V. As seen from Figure 5b, the G/n-Si with a graphene source from supernatant have highest on/off ratio, and those made of sediment have the lowest on/off ratio, which might be caused by impurities in the sediment. Similarly, the supernatant devices also have the highest rectification factors, while the junctions between sediment graphene and silicon exhibit lowest rectification factors, as seen in Figure 4. The ideality factor is derived from the slope of the dark *J-V* curve. The Shockley ideal diode equation gives the *J-V* characteristic of an ideal diode in either forward or reverse bias (or no bias). The equation is as follows:

(1) $$J=J_{S}(e^{V_{D}/nV_{T}}-1)$$

where *J* is the diode current, *J*_*S*_ is the reverse bias saturation current (or scale current), *V*_D_ is the voltage across the diode, *V*_T_ is the thermal voltage (*k*_*B*_T, *k*_*B*_ is the Boltzmann constant, *T* the temperature in Kelvin), and *n* is the ideality factor, also known as the quality factor, or sometimes emission coefficient. The ideality factor *n* varies from 1 to 2 depending on the fabrication process and the semiconductor material and, in many cases, is assumed to be approximately equal to 1. As shown in Figure 3, the ideality factor of the G/n-Si devices change in the order *n*(Su) > *n*(So) > *n*(Se) . The saturation current density *Js* can be expressed with thermionic emission model [20] in the form of current density (mA/cm^2^)

(2) $$J_{S}=AT^{2}e^{-e\varphi_{\mathrm{SB}}/k_{B}T}$$

where *A* is the Richardson constant, which is 112 A/(cm^2^K^2^)for n-Si and 32 A/(cm^2^K^2^) for p-Si substrates [21], and *eϕ*_SB_ is the zero-bias Schottky barrier height. By combining equations 1 and 2 and fitting the *J-V* curves of the devices in dark, the Schottky barriers are estimated to be 0.52 to 0.67 eV on average for the graphene/n-Si devices and 0.61 to 0.73 eV for the graphene/p-Si devices at 300 K, which is consistent with the higher current densities observed in n-Si devices versus p-Si devices. These values are comparable to the values of the bilayer graphene-on-silicon devices [22], but considerably smaller than the Schottky barrier heights (approximately 0.7 eV) measured previously in graphene/graphene oxide interfaces [23], and the value obtained from the temperature-dependent data (approximately 0.85 eV) [24].

Usually graphene flakes, generally known to exhibit weak p-type conductivity [25], and different Schottky junction solar cells composed of the graphene and n-/p-type Si substrates were evaluated (Figures 5a,b). By virtue of the formation of Schottky barrier, excellent rectification characteristics are observed (Figure 5a). Moreover, these devices exhibit pronounced photovoltaic effects upon white light illumination. As shown in Figure 5b, the device made of p-Si wafer shows the worst performance, with a power conversion efficiency of less than 0.005 %. In contrast, substantial increase of to 0.02 % is observed when graphene flakes are deposited onto n-type silicon wafers. Further increase in the conversion efficiency was observed for devices made of Se and Su graphene flakes. It might be due to the fact that Su graphene has much less impurities which could cause exciton quenching.

**Figure 6:**
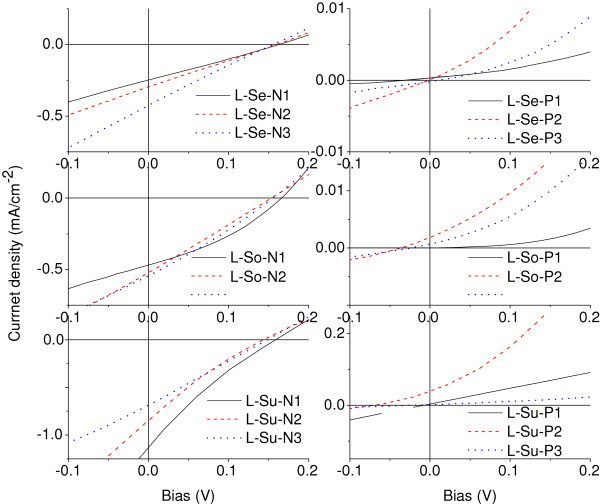
***J-V*characteristic curves of the G/n-Si (left) and G/p-Si (right) devices with different grapheme sources under AM1.5 illumination.**

**Figure 7:**
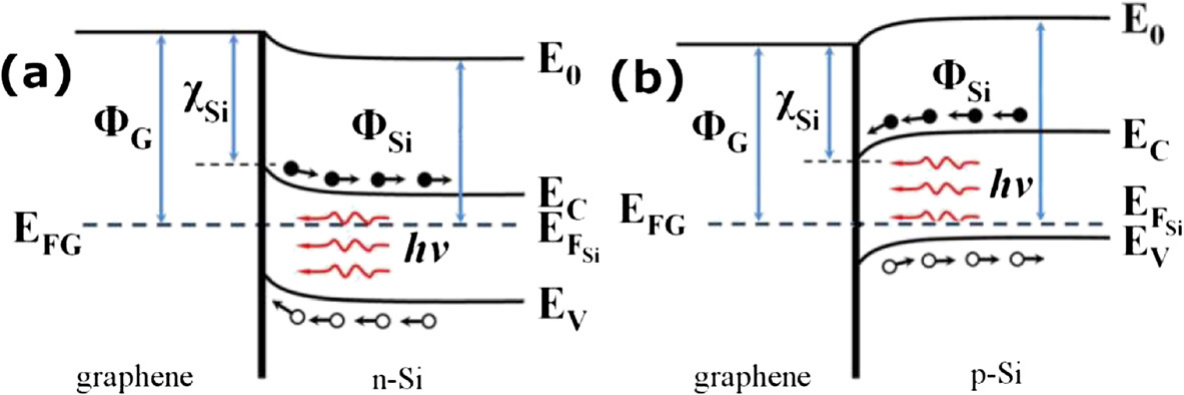
**Energy diagrams of G/n-Si and G/p-Si Schottky junctions upon light illumination.** The energy diagrams of G/n-Si (a) and G/p-Si (b) Schottky junctions upon light illumination, respectively. Φ_G_/Φ_Si_ and E_*F*G_/E_*F*Si_ denote the work functions and Fermi energy levels of graphene/Si. *χ*_Si_ is the electron affinity of silicon. E_*C*_ and E_*V*_ are the conduction band and valence band of silicon, respectively.

To investigate the underlying physics of the G/Si Schottky junction solar cells, photoresponse characteristics of both G/n-Si and G/p-Si devices were shown in Figure 6a,b. The observed high sensitivity to the light illumination, with a large i_on_/i_off_ ratio of >10^2^, suggests that the electron–hole pairs could be efficiently generated and separated in the G/Si solar cells, which helps facilitate the Schottky junctions to harvest solar light more effectively. The schematic energy band diagrams of G/n-Si and G/p-Si were displayed in Figure 7a,b. As a result of the formation of Schottky barrier at the G/Si interface, partial carriers in Si substrates tend to move to the graphene side and consequently, the energy levels near the Si surface will bend upward (for n-Si) or downward (for p-Si), causing the formation of space-charge region and built-in electric field near the G/Si interface. Upon light illumination, the photogenerated electron–hole pairs will be separated within the built-in field region, and the resulted free electrons and holes will move towards opposite directions, which results in the generation of photocurrent. This model suggests that the graphene films serve not only as transparent electrodes but also as important active layers in the devices.

Although the photovoltaic conversion efficiency of the G/Si solar cells is relatively low, the photovoltaic performance could be improved by doping graphene with nitrogen in G/p-Si configuration, which could be attributed to the enhanced n-type conductivity of the graphene film at higher N doping concentration, or treating graphene with a strong acid such as SOCl_2_ or nitric acid in G/n-Si devices [26]. Additionally, increasing the graphene flake size would be helpful for the improvement of photovoltaic conversion of both devices.

## Conclusion

We developed chemical vapor deposition approach to the synthesis of graphene at large scale and low cost, which makes the wide applications of graphene possible. The graphene coating on n-Si wafer forms Schottky junction with rectification behavior. The fabrication of Schottky junctions has the merits of low cost and simplicity. By comparing different graphene sources, it was found that the supernatant graphene is the most desirable material for the fabrication of G/n-Si junction with excellent rectifying capability and good photovoltaic conversion efficiency. Although G/p-Si also exhibit rectification behavior, they demonstrate poor photovoltaics conversion efficiency due to the weak p-type conductivity of our graphene flakes.

## Competing interests

The authors declare that they have no competing interests.

## Authors' contributions

MM and ZL contribute equally in this work. MM carried out the experiments and measurements; ZL offered idea, performed data analysis and drafted the manuscript. JC and CT participated in the discussion. All authors read and approved the final manuscript.

## Authors' information

MM is Ph.D. candidate connected with the Department of Physics and Astronomy, University of Arkansas at Little Rock. ZL is a visiting professor at the Department of Physics and Astronomy, University of Arkansas at Little Rock, JC and TC are the professors at Department of Physics and Astronomy, University of Arkansas at Little Rock.
